# ASSEMBLY LINE AILMENT: A TALE OF A DISCOLORED DIGIT IN THE AUTO INDUSTRY

**DOI:** 10.51894/001c.122890

**Published:** 2024-08-30

**Authors:** Konstantin Rosich, Christopher Nedzlek

**Affiliations:** 1 Emergency Medicine Henry Ford Wyandotte

## 34

### INTRODUCTION

This case report discusses a 45-year-old male presenting with acute symptoms of “freezing ring finger” and discoloration in the fourth finger of his left hand. The patient’s occupation at a Ford automotive factory involved prolonged and repetitive use of hand tools, raising concerns about occupational-related vascular complications. This report explores the diagnostic and therapeutic approach to identifying Hypothenar Hammer Syndrome (HHS) and highlights the importance of recognizing occupational risk factors.

### CASE DESCRIPTION

45-year-old male reported a 24-hour history of pain, numbness, and discoloration in the ring finger of his left hand, following prolonged use of hand tools. Clinical examination revealed a cold and pale fourth finger, indicating compromised blood flow. Despite intact range of motion, motor function, and sensation, CT angiography confirmed the diagnosis of hypothenar hammer syndrome (HHS), a vascular disorder resulting from repetitive trauma to the ulnar artery at the hypothenar eminence. Prompt intervention was initiated with Heparin infusion, and vascular surgery consultation recommended conservative measures. These included modification of work practices, hand protection, and dual antiplatelet therapy to prevent further thrombotic events. The patient was discharged with follow-up scheduled the next day.

### DISCUSSION/CONCLUSION

This case underscores the occupational risk factors associated with HHS in industrial settings, particularly in individuals engaged in repetitive hand-intensive tasks within the automotive manufacturing sector. The timely diagnosis and intervention with anticoagulation and conservative measures are crucial in preventing further complications and preserving hand function. Healthcare providers must be vigilant in recognizing the link between occupational exposures and vascular disorders, allowing for early intervention and preventive measures. A collaboration between occupational health, vascular specialists, and the affected worker can facilitate a comprehensive approach to manage and mitigate the impact of hypothenar hammer syndrome in the industrial workplace.

